# 3D simulation of percutaneous sustentaculum tali screw insertion in calcaneal fractures

**DOI:** 10.1186/s12891-023-06748-5

**Published:** 2023-08-08

**Authors:** Xian. Li, Xiao-ke. Wang, Lian-kui. Yu, Chao. Zhang, Ming-ming. Zhao, Jun. Yan, Li-ren. Han

**Affiliations:** 1 School of Clinical Medicine, Weifang Medical University, Weifang, China; 2 Department of Orthopaedic Surgery, Liaocheng People’s Hospital, Liaocheng, China

**Keywords:** Calcaneal fracture, Percutaneous screw fixation, Sustentaculum tali, Digital measurement

## Abstract

**Background:**

In calcaneal fractures, the percutaneous screw fixation (PSF) is currently considered to be the better choice, but it is difficult to accurately place the screw into the sustentaculum tali (ST) during the operation. In this study, the ideal entry point, angle, diameter and length of the screw were calculated by simulating the operation process.

**Methods:**

We retrospectively collected the calcaneus computed tomography (CT) scans of 180 adults, DICOM-formatted CT-scan images of each patient were imported into Mimics software to establish calcaneus model. Virtual screws were placed on the lateral of the posterior talar articular surface (PTAS), the lateral edge of the anterior process of calcaneus (APC), and the calcaneal tuberosity, respectively, the trajectory and size of the screws were calculated.

**Results:**

The mean maximum diameter of the PTAS screw was 42.20 ± 3.71 mm. The vertical distance between the midpoint of the APC optimal screw trajectory and the lowest point of the tarsal sinus was 10.67 ± 1.84 mm, and the distance between the midpoint of the APC optimal screw trajectory and the calcaneocuboid joint was 5 mm ~ 19.81 ± 2.08 mm. The mean maximum lengths of APC screws was 44.69 ± 4.81 mm, and the Angle between the screw and the coronal plane of the calcaneus from proximal to distal was 4.72°±2.15° to 20.52°±3.77°. The optimal point of the maximum diameter of the calcaneal tuberosity screw was located at the lateral border of the achilles tendon endpoint. The mean maximum diameters of calcaneal tuberosity screws was 4.46 ± 0.85 mm, the mean maximum lengths of screws was 65.31 ± 4.76 mm. We found gender-dependent differences for the mean maximum diameter and the maximum length of the three screws.

**Conclusions:**

The study provides effective positioning for percutaneous screw fixation of calcaneal fractures. For safer and more efficient screw placement, we suggest individualised preoperative 3D reconstruction simulations. Further biomechanical studies are needed to verify the function of the screw.

## Introduction

Calcaneal fractures are the most common traumatic injuries of the tarsal bones and approximately 75% of calcaneal fractures have a displaced intra-articular component [1]. Surgical treatment of calcaneal fractures can restore articular congruity and obtain optimal improvement of patient outcomes [2, 3]. However, the fractures involving intra-articular are challenging injuries to manage because of their complex nature, technical challenges, devastating complications and difficult rehabilitation [4]. Previous studies have shown that some minimally invasive surgical methods, such as balloon calcaneplasty, arthroscopically assisted reduction and fixation, and percutaneous screw fixation, not only have the same reduction quality and functional outcome as open reduction and internal fixation, but also can reduce the incidence of postoperative complications [2, 5]. At the same time, the percutaneous screw fixation (PSF) is a better option, which can not only provide a strong fixation, but also can minimize damage to the surrounding tissues, subsequently result in shorter operative time and hospital stay, and reduced intraoperative blood loss, postoperative pain, and complication rates, specially for Sanders II fractures and a few Sanders III fractures [3, 6–8]. Placement of the screw into the sustentaculum tali (ST) from the lateral calcaneus is essential. ST screw fixation can maintain anatomic reduction, stable fixation, and early range of motion of the posterior talar articular surface (PTAS) while preventing displacement of the fracture pieces [9, 10].

**Fig. 1:**
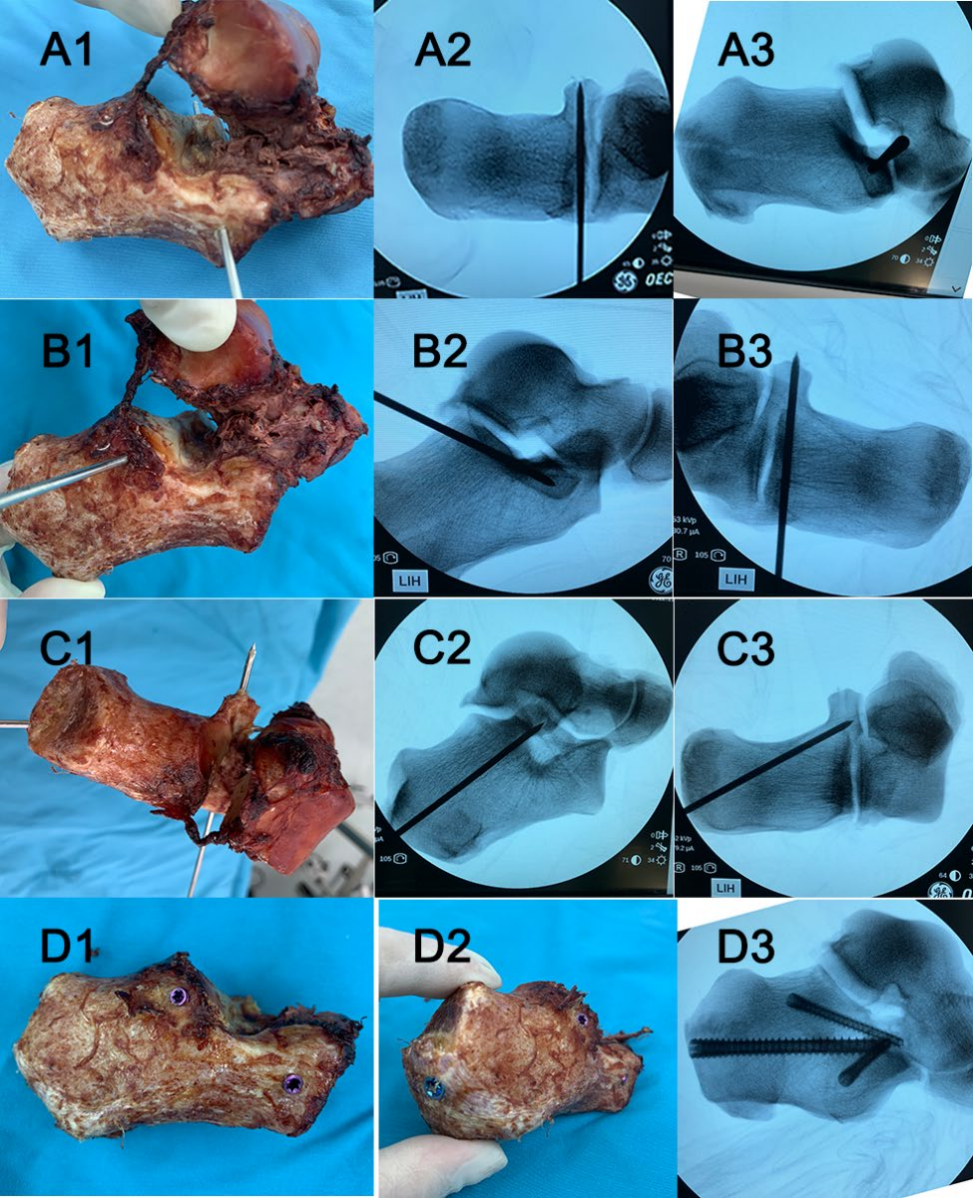
Three screws simulated on the calcaneal specimen and the screws X-ray perspective. (**A1-A3**) APC Kirschner wire trajectory. (**B1-B3**) PTAS Kirschner wire trajectory. (**C1-C3**) Kirschner wire trajectory of calcaneal tuberosity. (**D1-D3**) Three screws were placed in the calcaneal specimen

The major challenge of PSF for displaced intra-articular calcaneus fractures is placing screw that will maintain reduction by achieving fixation in the center of the ST without passing through the subtalar joint or the medial calcaneal cortex or affecting important medial structures. We simulated the placement of three screws into the ST on a calcaneal specimen from the lateral edge of PTAS(4 mm), the lateral edge of the anterior process of calcaneus (APC)(4 mm), and the calcaneal tuberosity(6 mm) (Fig. 1). There have been previous studies on ST screws, but some lack of discussion on screw trajectory and size [11–17].

The purpose of the study is to specify the ideal insertion point, the largest secure diameter, length and the accurate angle of the calcaneal screw through the method of 3D simulation.

## Materials and methods

We retrospectively collected the calcaneus CT scans of 180 adults who had undergone continuous slice CT scanning at the imaging research center of our hospital during January 2017 and July 2022, including 80 females and 100 males. Patients were excluded if they had deformity, fracture, arthritis, or tumor in the foot. This study was conducted in accordance with the World Medical Association Declaration of Helsinki and approved by the Ethical Committee of Liaocheng People’s Hospital. Informed consent was obtained from the patient who participated in this study. All methods were carried out in accordance with relevant guidelines. The mean age of the patients on whom the models were based was 45.08 ± 10.32 years (range18–88years).

DICOM-formatted CT-scan images of each patient were imported into Mimics software (21.0;Materialise, Leuven, Belgium). We removed the soft tissue and other tarsal bones by the function of image segmentation, region growth and multiple slice editing of Mimics software, respectively. A total of 180 virtual calcaneus models were created.

**Fig. 2:**
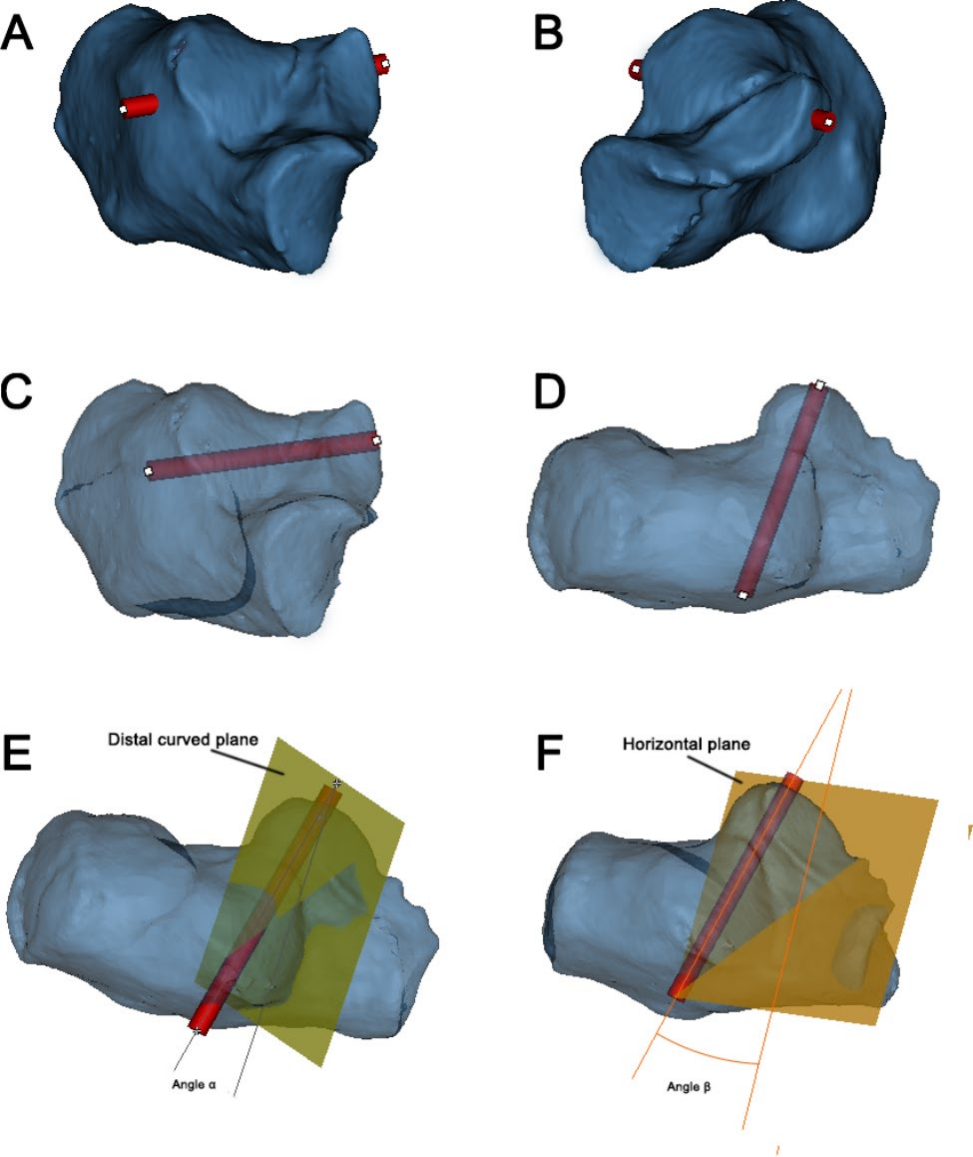
The measurement of virtual PTAS screw in the model. (**A, B**) Observed from the lateral and medial of the opaque 3D model,respectively. The screws did not penetrate the cortical bone. (**C, D**) Adjusted to the optimal length of the screw from the translucent 3D model. (**E**) Angle α: subtalar convergence angle. (**F**) Angle β: anteversion angle

**Fig. 3:**
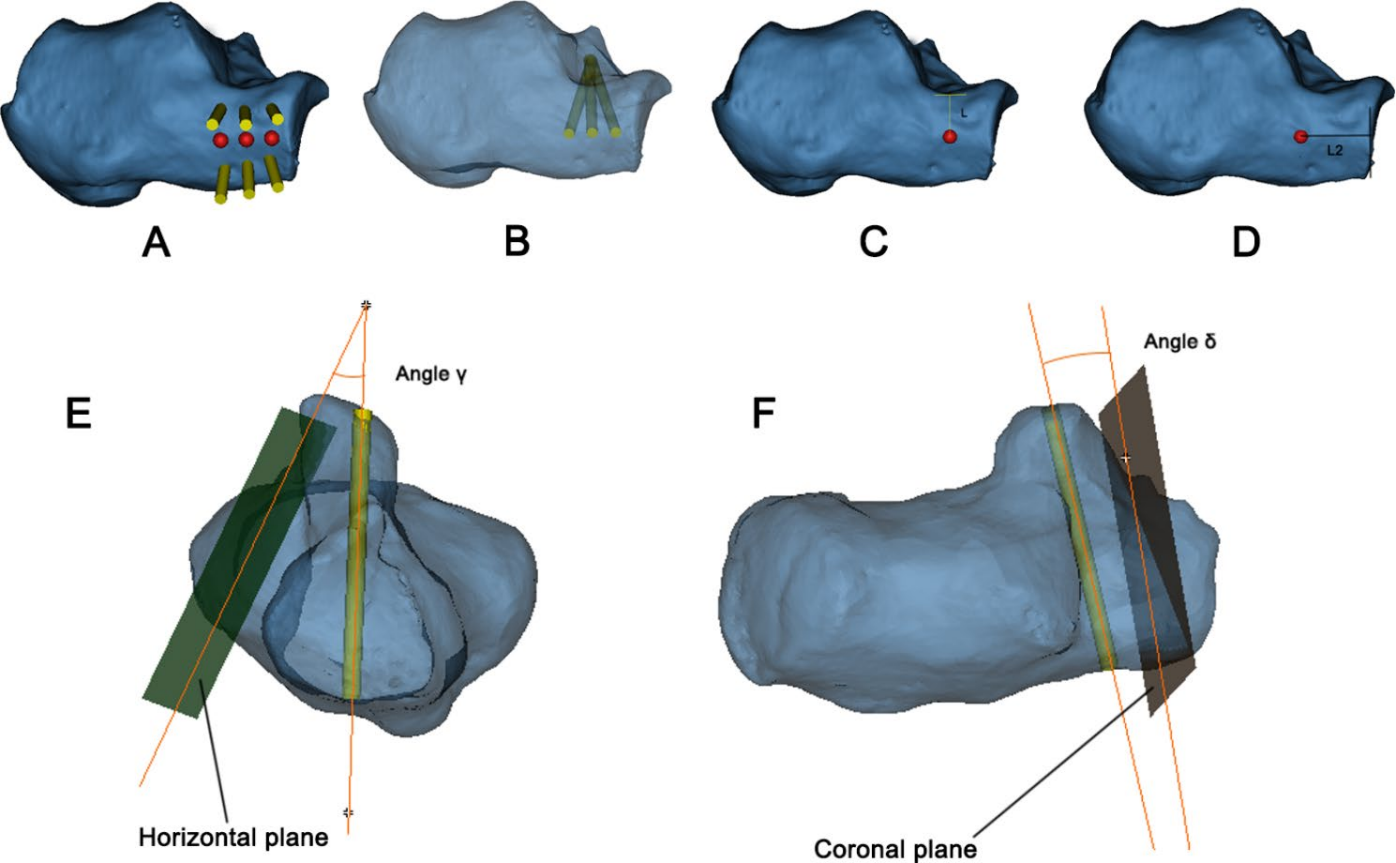
The measurement of virtual APC screws in the model. (**A**) The entry points of six screws were the most proximal and the most distal safe positions. The red points in the middle of the screws was the ideal entry point. (**B**) Three screws were placed at the optimal entry point. (**C**) L_1_: The perpendicular distance from the optimal entry point to the lowest point on the lateral margin of the sinuses tarsi. (**D**) L_2_: The length of the calcaneocuboid joint surface to the midpoint of the farthest insertion trajectory. (**E**) Angle γ: anteversion angle between the APC screw and the horizontal plane of the calcaneus. (**F**) Angle δ: the angle between the APC screw and the coronal plane of the calcaneus

We reduced the transparency of the calcaneus models and the length of the PTAS screw was continuously adjusted to ensure that the screw did not penetrate the bone cortex (Fig. 2A-D). The distal curved surface of the posterior articular surface was selected as the reference plane and marked as the plane (1) The subtalar convergence angle between the PTAS screw and plane 1 was measured and recorded as angle α (Fig. 2E). Then the horizontal plane of the calcaneus was selected and marked as plane (2) The anteversion angle between the PTAS screw and plane 2 was measured and recorded as angle β (Fig. 2F). After that, lateral to the APC, inferior to the distal end of the PTAS, and 5 mm distance to the articular surface of distal calcaneus, sinuses tarsi and cuboid articular surface, six ST screws were sequentially placed from distal to proximal (Fig. 3A). The channel between the screws is safe for the entry track and the midpoint of the proximal and distal safe channel was considered the ideal insertion point, because this position allowed for the optimal track access to the center of the ST (Fig. 3B). Meanwhile, the perpendicular distance from the midpoint to the nadir of the lateral sinuses tarsi was measured and recorded as L_1_ (Fig. 3C). The length of the calcaneocuboid joint surface to the midpoint of the farthest insertion trajectory was recorded as L_2_(Fig. 3D). The anteversion angle between the APC screw and plane 2 was measured and recorded as angle γ (Fig. 3E). Then the coronal plane of the calcaneus was selected and marked as plane 3 and the angle between the APC screw and plane 3 was measured and recorded as angle δ (Fig. 3F).

**Fig. 4:**
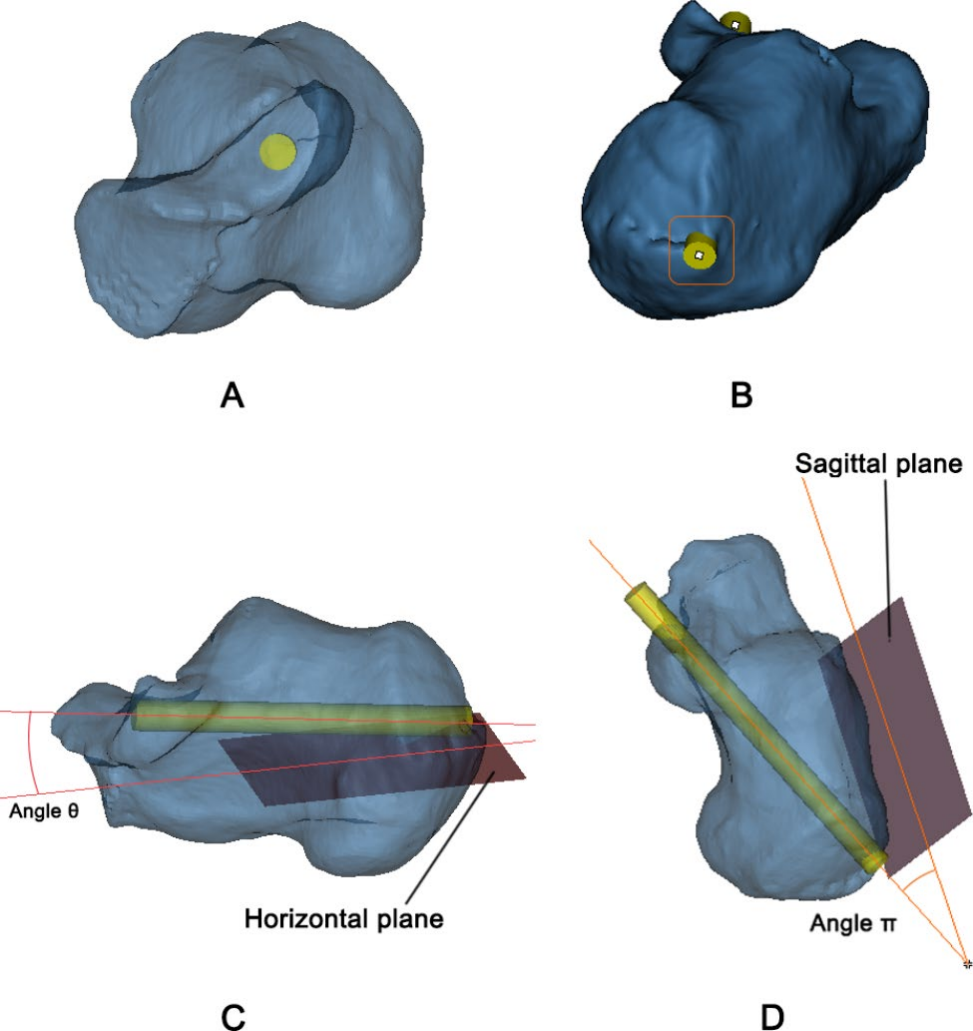
The measurement of virtual calcaneal tuberosity screw in the model. (**A**) Adjusted to the optimal lengths and diameters of the screws from the translucent calcaneus model. (**B**) The lateral border of calcaneal tuberosity was the optimal entry point. (**C**) Angle θ: the angle between the screw and the horizontal plane of the calcaneus. (**D**) Angle π: the angle between the screw and the sagittal plane of the calcaneus

Finally, the calcaneus model was adjusted to a semi-transparent state, and axial fluoroscopy was converted to observe and measure from ST to different positions of the calcaneal tuberosity to search for the largest translucent area (Fig. 4A). We found that the center point of the largest translucent area was located at the lateral border of the achilles tendon endpoint (Fig. 4B). The virtual screw was placed in the center of the translucent area and adjusted continuously to find the maximum diameter, which was defined as the maximum diameter when the screw did not penetrate the bone cortex. Meanwhile, the length of the screw was constantly adjusted to ensure that the screw just penetrated the bone cortex. The angle between the calcaneal tuberosity screw and plane 2 was measured and recorded as angle θ (Fig. 4C). The sagittal plane of the calcaneus was selected and marked as plane 4 and the angle between the screw and plane 4 was measured and recorded as angle π (Fig. 4D).

The collected data were analysed by SPSS 25.0 statistical software. The experimental data are represented as the mean ± SD. T tests were used to compare the data. Statistical significance was accepted at P < 0.05.

## Results

The study subjects included 100 males and 80 females aged between 18 and 88 years old, with a mean age of 45.08 ± 10.32 years.

The midpoint of the lateral edge of the PTAS was the optimal entry point, the perpendicular distance from the midpoint to the posterior articular surface was 10.78 m [16]. As shown in Fig. 2A, B, virtual screw was inserted from the PTAS into the ST in the reconstructed calcaneus model. As shown in Table 1, the mean maximum lengths of PTAS screws was 42.20 ± 3.71 mm. For the data captured above, the intersex difference was significant (P < 0.05). The mean angle α was 14.41°±4.42°and the angle β was 9.31°±3.07°. Obviously, the results were not statistically significant between males and females(P > 0.05).

**Table 1:** Comparison between different genders: Lengths of PTAS screws, Angle α and β

Group	Length^y^(mm)	α(°)	β(°)
All(n = 180)	42.20 ± 3.71	14.41 ± 4.42	9.31 ± 3.07
Male(n = 100)	44.27 ± 3.29	14.35 ± 4.69	9.43 ± 3.27
Female(n = 80)	39.90 ± 2.72	14.48 ± 4.38	9.18 ± 3.02
t value^x^	3.170	-0.065	0.173
P value^x^	0.006	0.950	0.866

^x^t and P are the results of gender comparisons

^y^For the Lengths, Angle α and β, intersex difference was significant (P < 0.05)

Then as shown in Table 2, the mean maximum lengths of APC screws was 44.69 ± 4.81 mm and the mean distance of L_1_ (the perpendicular distance from the midpoint to the nadir of the lateral sinuses tarsi)was 10.67 ± 1.84 mm, L_2_ was 19.81 ± 2.08 mm, the distance between the midpoint of the APC optimal screw trajectory and the calcaneocuboid joint was 5 mm ~ 19.81 ± 2.08 mm. For the data captured above, the intersex difference was significant (P < 0.05). As shown in Table 2, The mean angle γ was 14.41°±4.42°and the angle δ from proximal to distal was 4.72°±2.15° to 20.52°±3.77°. The results were not statistically significant between males and females(P > 0.05).

**Table 2:** Comparison between different genders: Average lengths of APC screws, L_1_, L_2_, Angle γ and δ

Group	Length^y^(mm)	L_1_^y^(mm)	L_2_^y^(mm)	γ(°)	δ-proximal(°)	δ-distal(°)
All(n = 180)	44.69 ± 4.81	10.67 ± 1.84	19.81 ± 2.08	19.59 ± 4.06	4.72 ± 2.15	20.52 ± 3.77
Male(n = 100)	47.31 ± 4.12	11.49 ± 1.51	20.82 ± 1.56	20.43 ± 3.83	4.73 ± 2.10	20.54 ± 3.77
Female(n = 80)	41.19 ± 3.86	9.76 ± 1.80	18.30 ± 1.92	18.66 ± 4.34	4.70 ± 2.37	20.49 ± 3.99
t value^x^	3.016	2.288	-2.807	0.938	0.036	0.025
P value^x^	0.008	0.035	0.015	0.362	0.972	0.951

^x^t and P are the results of gender comparisons

^y^For the Lengths and the distance of L_1_, L_2_, intersex difference was significant (P < 0.05)

Through the axial perspective, we found that the center point of the largest translucent area was located at the lateral border of the achilles tendon endpoint (Fig. 4B). From Table 3, the mean maximum diameters of calcaneal tuberosity screws was 4.46 ± 0.85 mm, the mean maximum lengths of screws was 65.31 ± 4.76 mm. For the data captured above, the intersex difference was significant (P < 0.05). In addition, the mean angle θ was 8.03°±3.50°and the mean angle π was 31.94°±4.30°, but the results were not statistically significant between males and females(P > 0.05).

**Table 3:** Comparison between different genders: Diameters of screws, Lengths of screws, Angle θ and π

Group	Diameter^y^(mm)	Length^y^(mm)	θ(°)	π(°)
All(n = 180)	4.46 ± 0.85	65.31 ± 4.76	8.03 ± 3.50	31.94 ± 4.30
Male(n = 100)	4.72 ± 0.77	67.78 ± 3.38	7.91 ± 3.73	32.62 ± 4.72
Female(n = 80)	4.06 ± 0.82	61.61 ± 4.21	5.20 ± 3.46	30.91 ± 3.73
t value^x^	2.735	3.146	-0.150	0.743
P value^x^	0.009	0.008	0.883	0.470

^x^t and P are the results of gender comparisons

^y^For the Diameters, Lengths, Angle θ and π, intersex difference was significant (P < 0.05)

The safety tracks of PTAS and APC allowed 3.5 and 4 mm screws to be inserted into the ST, and the maximum safe diameter of the calcaneal tuberosity screw is 4 mm.

## Discussion

In PSF, ST screw can maintain a firm and stable fixation, but it is difficult for us to accurately place the screw into the ST [9, 10]. Liao et al. and Phisitkul et al. found the optimal entry point and Angle of the PTAS screws, Liao et al. calculated the perpendicular distance from the PTAS screw to the posterior articular surface was 10.78 m, Phisitkul et al. calculated the subtalar convergence and anteversion angles of the screws [15, 16]. However, the double bended structure of the posterior articular surface should be considered during screw placement. Then we re-selected the distal curved surface of the posterior articular surface as the reference plane to determine the subtalar convergence angle. In this study, we did not adjust the PTAS screw to other positions, because Liao et al. found that the midpoint of the lateral edge of the PTAS was the optimal entry point, and fixing the screw at the midpoint had sufficient force to prevent fracture fragment displacement, and it is easier to determine the anatomical position during the operation. From Table 1, the results showed (42.20 mm) that the length of PTAS screw was 0.77 mm different from that of Liao Liqing’s(42.97 mm) [16]. In our research, the lengths of the PTAS screws were significantly larger in males compared with females. This may be due to anatomical differences in the calcaneus between female and male. However, the mean angle α and β between male and female had no statistical criteria. From Table 1, a smaller Angle of PTAS screw can be used to achieve ST.

**Fig. 5:**
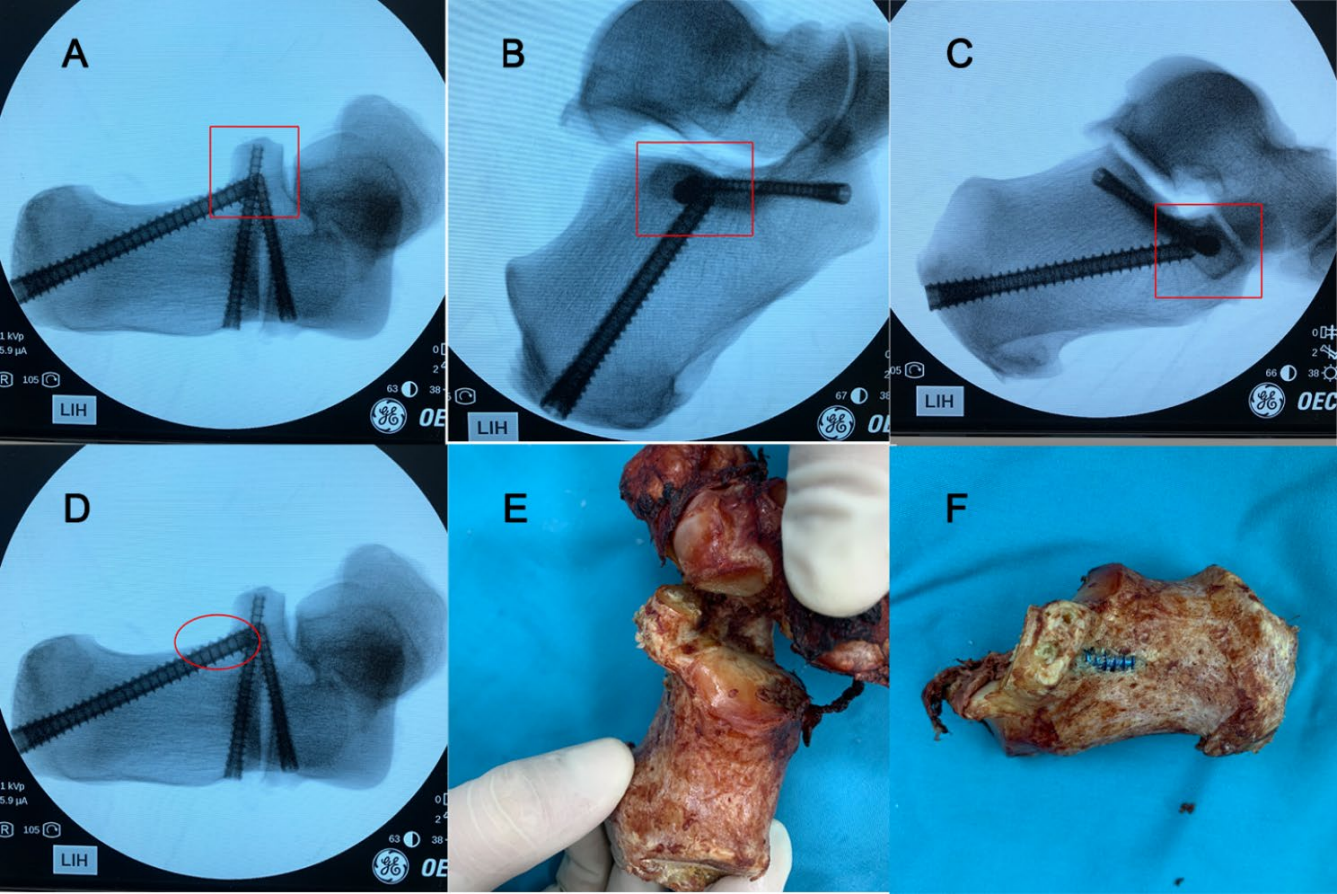
Axial views of the calcaneus and vertical views of the screws. (**A**) Axial view of the calcaneus. (**B**) Vertical view of the PTAS screw. (**C**) Vertical view of the APC screw. (**D**) Axial view of the calcaneus shows the screw breaking through the medial bone cortex. (**E, F**) The screw penetrates the bone cortex in the calcaneal specime

In addition, Gitajn et al. used axial Harris heel views and Geerling et al. used 3D imaging techniques to evaluate the accuracy of PTAS screw placement [12]. However, at the axial view of the calcaneus, it is not possible to accurately assess whether the screw is in the ST or breaks through the articular surface(Fig. 5A). Olexa et al. studied the ST axial view, but were also unable to determine whether the screw penetrated the ST or PTAS [18]. We found that when the view was perpendicular to the screw from the lateral aspect of the calcaneus, it was clear that the screw was located in the center of the overlap shadow between the ST and the subarticular bone (Fig. 5B). By combining axial views, it is sufficient to verify whether the screw is within the ST or penetrates the bone cortex. Although 3D imaging technology can better visualize and identify articular reduction and screw placement, it requires high infrastructure and operating environment, which needs further improvement to make the surgical process more convenient. Meanwhile, Gras et al. introduced different navigation methods for the ST screw and demonstrated that the 2D navigation technique was more reasonable during surgery [13]. Therefore, experienced surgeons can successfully complete the placement of PTAS screws under the C-arm fluoroscopy.

APC can involve various fracture types, and its fixation affects foot function [14]. Most of the previous studies on APC screw placement were for open reduction and internal fixation, but few were for ST placement [3, 4, 10, 14]. The study by De Boer et al. showed that the use of Screw Targeting Clamp may have facilitated screw placement, but the required length of the screw could not be determined [19]. Therefore, we calculated the trajectory and required length of the APC screw. During the operation, the surgeon can make the screw perpendicular to the fracture surface into ST as much as possible under the C-arm fluoroscopy, so as to ensure the maximum force on the fragments of fracture and prevent the fracture block from displacement. As shown in Table 2, the lengths of the APC screws, the perpendicular distance from the midpoint to the nadir of the lateral sinuses tarsi and the length of the calcaneocuboid joint surface to the midpoint of the insertion trajectory were significantly larger in males compared with females. According to previously published guidelines, to prevent screw entry into the articular surface or destruction of cortical bone, the safe insertion point should be more than 5 mm away from the articular surface or lateral border of the calcaneus [20]. APC has a wide sufficient safety channel to allow 3.5 or 4 mm screws to reach the ST. Similarly, we found that the axial view of the calcaneus combined with the vertical view of the APC screw could also confirm whether the screw was in the ST or penetrating the bone cortex (Fig. 5C). This allows the surgeon to place the screws more accurately in the ST with the assistance of Screw Targeting Clamp.

Long et al. provided a reliable and reproducible protocol for PSF treatment of displaced intra-articular calcaneal fractures, but calcaneal tuberosity screws positioning and dimensions were not studied [17]. We found the maximum diameter of the screw in the translucent region of the axial view, and also identified the lateral border of the achilles tendon endpoint as the optimal insertion point. From Table 3, the diameter and length of the calcaneal tuberosity screw were significantly larger in males compared with females. From the axial view of the calcaneus, we could easily tell whether the screw is within the ST or penetrated the medial calcaneus bone cortex (Fig. 5D). When we placed a 6-mm screw in the calcaneal specimen, it was not difficult to see in the axial view that the screw penetrated the medial calcaneal cortical bone (Fig. 5D-F). According to the data, the maximum safe diameter of calcaneal tuberosity screw is 4 mm in both male and female. Of course, under C-arm fluoroscopy, the surgeon can also choose a larger diameter screw without passing through the subtalar joint or medial calcaneal cortex according to individual differences. Calcaneal tuberosity screws are longer relative to PTAS and APC screws, and the experience of the surgeon may be more demanding.

After screw placement, multi-directional perspective is required to ensure that the screw is as close to the articular surface as possible without penetrating. If screw penetrates the articular surface, the insertion point and appropriate Angle need to be re-selected. If there is a navigation system, the effect is better, and it is closer to the articular surface to obtain optimal holding force, so as to prevent the fracture fragment from shifting.

The parameters of the three screws may provide the surgeon with appropriate information on safe screw placement for PSF treatment of calcaneal fractures, so as to complete the operation more quickly and effectively. The large standard deviation of our results indicates great differences among individuals. As a result, preoperative planning should be implemented in detail for each patient. 3D reconstruction and simulated screw placement techniques with digital software before surgery are valuable.

There are still some limitations to this study. We only analyzed the data based on genders, not different age, body size and type of fracture groups. These factors may also affect screw placement and fracture block stability. We only studied the calcaneus of Chinese people, who have different skeletal shapes with American and European populations. In addition, the study was performed on intact calcaneus, but the normal anatomy is distorted in the case of calcaneal fractures. It is necessary to improve the quality of fracture reduction by preoperative 3D reconstruction and intraoperative use of reduction forceps. Thus, the effect of ST screw and other calcaneal screws needs to be verified by more biomechanical research and related clinical research.

## Conclusion

We provided guidelines for PSF of calcaneal fractures in a 3D simulation. In the 3D model, we positioned and measured the dimensions of the three screws. Data from simulated screws need to be combined with clinical experience for safer placement of screws into the ST. Further biomechanical tests and clinical studies are needed to verify its effects.

## Data Availability

The datasets generated and analyzed during the current study are available from the corresponding author on reasonable request.

## Abbreviations

3-D: Three-dimensional
CT: Computed tomography
DICOM: Digital Imaging and Communication in Medicine
Mimics: Materialise’s Interactive Medical Image Control System
SPSS: Statistical Package for the Social Sciences
SD: Standard deviation
PSF: The percutaneous screw fixation
ST: The sustentaculum tali
PTAS: The posterior talar articular surface
APC: The anterior process of calcaneus
